# Trends and characteristics of enrolment in the National Health Insurance Scheme in Ghana: a quantitative analysis of longitudinal data

**DOI:** 10.1186/s41256-018-0087-6

**Published:** 2018-11-13

**Authors:** Eric Nsiah-Boateng, Moses Aikins

**Affiliations:** School of Public Health, College of Health Sciences, University of Ghana, Accra, Ghana

**Keywords:** Trends, Enrolment, Longitudinal data, National Health Insurance Scheme, Ghana

## Abstract

**Background:**

In 2004, Ghana started experimenting a National Health Insurance Scheme (NHIS) to reduce  out-of-pocket payment for healthcare. Like many other social health insurance schemes in Africa, the NHIS is striving for universal health coverage (UHC). This paper examines trends and characteristics of enrolment in the scheme to inform policy decisions on attainment of UHC.

**Methods:**

We conducted trend analysis of longitudinal enrolment data of the NHIS for the period, 2010–2017. Descriptive statistics were used to examine trends and characteristics of enrolment by geographical region and member groups.

**Results:**

Over the 8-year period, the population enrolled in the scheme increased from 33% (8.2 million) to 41% (11.3 million) between 2010 and 2015 and dropped to 35% (10.3 million) in 2017. Members who renewed their membership increased from 44% to 75.4% between 2010 and 2013 and then dropped to 73% in 2017. On average, the urban regions had significantly higher number of new enrolments than the rural ones. Similarly, the urban and peri-urban regions recorded significantly higher number of renewals than the other regions. In addition, persons below the age of 18 years and the informal sector workers had significantly higher number of enrolment than any other member group.

**Conclusions:**

Enrolment in the NHIS is declining and there are significant differences among geographical regions and member groups. Managers of the NHIS need to enforce the mandatory enrolment provision in the Act governing the scheme, employ innovative strategies such as mobile phone application for registration and renewals and address delays in healthcare provider claims to improve enrolment.

**Electronic supplementary material:**

The online version of this article (10.1186/s41256-018-0087-6) contains supplementary material, which is available to authorized users.

## Background

Over the last two decades, the concept of social health insurance (SHI) has gained attention in social policy development discourse [1–3]. SHI is seen as the most sustainable healthcare financing model for providing financial risk protection for majority of the population in low and middle-income countries (LMICs) [4, 5]. The SHI concept has become more imperative following the World Health Assembly’s call for universal health coverage (UHC) in health systems in 2005 [1, 6] and the inclusion of UHC in the UN Sustainable Development Goals (SDGs) in 2015. The SDG goal 3, target 8, mandates member countries to “Achieve universal health coverage, including financial risk protection, access to quality essential healthcare services and access to safe, effective, quality and affordable essential medicines and vaccines for all” by the year 2030 [7, 8]. The overarching goal of UHC is that in an event of illness or sickness, all people will have access to the quality, essential health service they need without being exposed to financial hardship [2, 7].

Many African countries particularly those in the sub-Saharan Africa (SSA) are at different stages of SHI implementation aimed at achieving UHC [9, 10]. It is a general belief that UHC is a panacea for reducing out-of-pocket payment and inequity in access to and utilization of healthcare services [6, 9]. One distinct characteristic for SHI attractiveness in the sub-region is that it does not depend exclusively on public finance, but instead shares the responsibility of health financing among the population [11, 12].

Ghana, a lower middle-income country in SSA, implemented a National Health Insurance Scheme (NHIS) in 2004 to provide financial risk protection for all residents [13–17]. It was fashioned out to address the challenges of the out-of-pocket payment system of paying for healthcare services in the 1990s, popularly referred to as “cash and carry”. The cash and carry system was introduced at that time to recover at least 15% of recurrent expenditure and drugs [13, 18, 19] but it led to increased inequality in healthcare access and preventable deaths [10, 14, 18, 20]. The NHIS is managed by the National Health Insurance Authority (NHIA), a regulatory body mandated by law to oversee operations of public and private health insurance schemes in the country [21]. Since its implementation, the NHIS has made gains in population coverage, increased access to healthcare services by reducing out-of-pocket payment and contributed to raising revenue of healthcare providers [10].

At present, the NHIA has 159 district offices and five satellite offices across the 10 administrative regions of the country. It also has over 4000 network of public and private healthcare providers nationwide, rendering services under the minimum benefit package to card-bearing members [22]. The NHIS reportedly covers 95% of disease conditions afflicting the population [14, 21] and the benefit package is same for all card-bearing members. Broadly, it covers general consultation and medicines at the outpatient and inpatient departments; minor surgeries; admissions at the general wards; maternal care services; dental services; ear, nose and throat (ENT) services; and all emergency services. It, however excludes preventive services provided by the Ministry of Health, for example, immunization [14]. Services that have the potential to threaten sustainability of the scheme, for example, surgeries other than road traffic accidents and non-essential health services such as cosmetic surgeries are also excluded from the benefit package [14, 20, 21]. The NHIS is largely tax-financed through a 2.5% valued-added tax (VAT) on selected goods and services [17, 20, 21]. Other sources of funding include two and a half percentage point deductions from the formal sector workers social security contributions, premium from the informal sector workers, internally generated funds from activities of the scheme and donor support from development partners [20, 21].

Membership of the scheme is mandatory, but enforcement has been challenging, making enrolment practically voluntary. Members are broadly categorized into exempt and non-exempt groups. The exempt groups comprise those who do not pay premium to the scheme; persons below the age of 18 years, elderly aged 70 years and above, indigents (extreme poor), Social Security and National Insurance Trust (SSNIT) pensioners and beneficiaries of the Livelihood Empowerment Against Poverty (LEAP) programme. The formal sector workers or contributors of SSNIT are also exempted because their premium is income-rated and deducted at source. Following implementation of the free maternal healthcare policy in July, 2008 [23], pregnant women are also added to exempt group. However, all members of the exempt group with the exception of indigents, LEAP beneficiaries and pregnant women, pay registration and renewal fee of GHS8.00 ($1.18) and GHS5.00 ($1.13), respectively. The non-exempt groups are those who pay premium directly to the scheme, and they are the workers in the informal sector of the economy. In addition to the premium, the informal sector workers also pay registration and renewal fees. By design, the premium is graduated from GHS7.20 ($1.62) to GHS48.00 ($10.83) based on income levels of the members. However, due to lack of data on income levels, particularity for the informal sector workers, a flat premium is charged during registration but varies from GHS15.00 ($3.39) in the rural areas to GHS22.00 ($4.97) in the cities.

There are several studies on enrolment in the NHIS, particularly population coverage, renewal of membership and equity [17, 24–31]. However, the settings and data of these studies are limited. A study that examined enrolment by member groups was limited to one region and found significant differences among them [31]. Another study that examined enrolment by geographical region used data from literature for only two periods, 2005 and 2008 and found variations across the regions [17]. What is lacking in literature so far, is longitudinal analysis of a national level enrolment data of the NHIS to examine trends and characteristics by geographical region and member groups. The present study seeks to fill this gap by analysing population coverage, membership retention and growth rates over an 8-year period. This study focuses on one of the three dimensions of UHC-population coverage [7, 32]. We believe that findings of this study would inform policy decision-making to improve enrolment, revenue and risk pooling of the NHIS. The study would also serve as evidence for LMICs implementing SHI programmes.

### Conceptual framework for the study

**Fig. 1:**
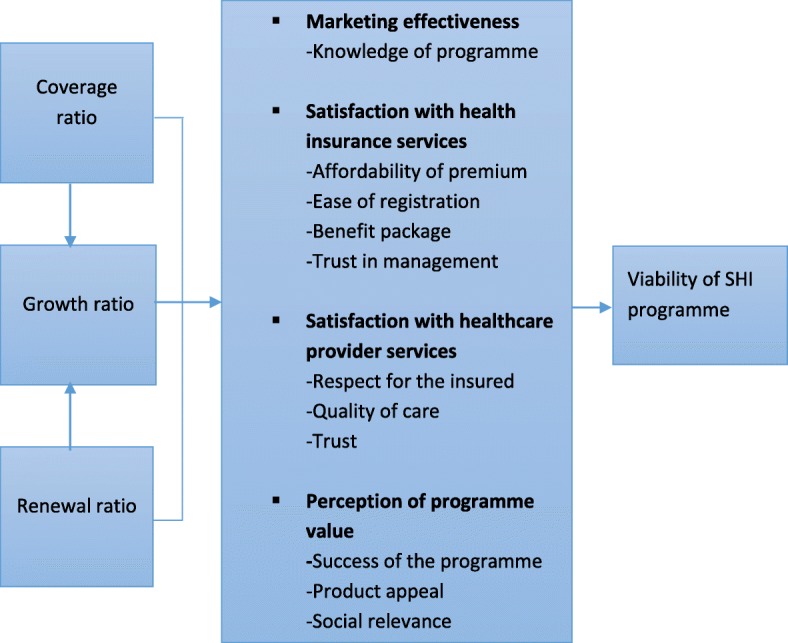
Conceptual framework for assessing viability of social health insurance programme. Source: Adapted from Wipf & Garand [33]

Although the study focuses on trends and characteristic of enrolment, it is situated in the Wipf and Garand framework for assessing product awareness and client satisfaction with life and SHI programmes [33]. According to the framework, the coverage ratio, renewal ratio and growth ratio are the three key indicators for assessing performance of health insurance schemes where enrolment is voluntary (Fig. 1). These indicators are important determinants for long-term viability of micro-insurance and SHI programmes with voluntary enrolment because they indicate how readily the target population enrol in the programme and retain coverage.

The coverage ratio measures proportion of the target population participating in the programme, serving as a key indicator of marketing effectiveness and programme’s success [33]. The marketing effectiveness, however depends largely on client’s satisfaction with the services and the perceived value of the programme [30, 33–36]. Voluntary enrolment of large proportion of the target population, particularly in SHI programmes, gives an indication of acceptance of the risk-pooling concept and understanding of the programme including how to access the benefits. Usually, a very low coverage ratio is an indication of adverse selection, where majority of the sick enrol in the programme.

The renewal ratio measures the proportion of insured that stay enrolled in the programme after their coverage term expires. It also gives an indication of the programme’s marketing performance and how satisfied the insured are. A very high renewal ratio, such as 90% or more, signifies that 1) there is a good understanding of the needs of the target population; 2) the price is acceptable to the target population; 3) the service levels are reasonable; and/or 4) the benefit is highly valued by the community [33]. On the other hand, low renewal ratio is an indication of client dissatisfaction, probably due to poor education and sensitization, poor services at healthcare provider facilities, unacceptable product value and unsatisfactory claims payment such as lengthy delays [24, 30, 33, 37]. A low renewal ratio could also mean that the insured does not know how and where to renew. It is recommended that a renewal ratio of at least 85% should be set as a minimum standard for insurance schemes with voluntary participation [33].

The growth ratio, which is the ratio of increase in the number of clients, measures how fast the number of clients in the programme is increasing or decreasing. Thus, it depends on the rate of coverage and renewal in the SHI programme. Generally, it increases at the initial stages of implementation of health insurance programmes but begins to decline as participation reaches its peak. A positive trend of the growth rate often indicates marketing success and product appeal. It also indicates social relevance of the programme to the target population. However, a declining growth rate indicates a loss of value and better alternative risk protection options [33]. It is suggested that for SHI programmes to remain viable from medium to long term, at least a zero growth rate should be maintained [33].

## Methods

### Study design and setting

This study is a trend analysis of longitudinal data of NHIS members for the period, 2010–2017. Although the NHIS became fully operational in 2005, it began to collect accurate routine data in 2010 [20, 38]. The scheme also started to disaggregate its enrolment data into new members, renewal and member groups in that same year. The study used enrolment data from all the 10 administrative regions of Ghana. In the last Population and Housing Census (PHC), the country had a population of 24.7 million with an average annual intercensal growth rate of 2.5% (see Additional file 1). The most populated region was Ashanti with a population of 4.8 million persons (19.4%) and the least was Upper East with population of 702,110 persons, representing 2.8% of the total population [39]. Population by density indicated that the Greater Accra Region was the most densely populated with approximately 1236 persons per square kilometre, followed by the Central region, 224 persons per square kilometre. The lowest densely populated region was the Northern region with a population of 35 persons per square kilometre.

Distribution of the population by sex showed that females constituted 51.2% (12.6 million) and this predominance occurred in all the regions except Western, where the number of males was approximately equal to females. Ghana’s population is predominantly youthful: persons aged less than 18 years constituted 44.7% (11.0 million), 18–59 years constituted 48.6% (11.9 million), and 60 years and above represented 6.7% (1.6 million). The dependent population (less than 15 and 65+ years) represented 43% (10.6 million). Furthermore, 55.3% of the population were economically active, of which 93.1% were employed. Among the economically active population, 93% are in the private sector and 64.8% are self-employed. There are high incidence of poverty in the predominantly rural regions including Upper East, Upper West, Northern, Western, Central and Volta compared to the more urbanised regions such as Greater Accra and Ashanti [40, 41].

Ghana has a decentralised public health system structure with healthcare facilities in all districts of the country [42, 43]. The healthcare facilities range from Community-Based Health Planning and Services (CHPS) compound to hospitals. Private and faith-based healthcare facilities complement the public healthcare facilities by providing about 40% of healthcare services to the population [42]. The private facilities are mainly located in the urban areas and largely patronised by the rich due to the perceived good quality of service and relatively high fees [43]. The faith-based facilities, however are mainly concentrated in the rural areas where government or public healthcare facilities are limited. There are predominantly large numbers of healthcare facilities and professionals in the urbanised regions of the country [44, 45].

### Study population and data collection

Enrolment data of members who enrolled or renewed coverage in the NHIS between 2010 and 2017 period were used for the study. The members comprised persons aged 18 years or above. The enrolment data were requested from the NHIA using a predefined data collection template. To ensure anonymity, the data request sheet contained no personal identifiers such as names, place of residence and telephone numbers of the members. We requested aggregated data across years of enrolment, geographical regions and defined member groups. First, the data obtained was cleaned of errors to ensure validity and completeness. Incomplete information such as unknown region and member group were verified from the Actuary Directorate of the NHIA. However, no observation was dropped from the dataset. A total of 78.6 million member-data were available for analysis.

### Data analysis

The membership data were analysed based on three key programme awareness and client satisfaction indicators, namely; coverage ratio, renewal ratio and growth ratio [33]. The *coverage ratio* was estimated as the number of active members divided by the target population, calculated for each year of the study period. Geometric population estimation method was used to estimate the target population for each year using the 2010 population census figure of 24,658,823 and average annual growth rate of 2.5%. The *renewal ratio* was calculated as the number of renewals (members that renewed their coverage) divided by the number of potential renewals (members eligible to renew) for each year of the study period. The *growth ratio* was calculated as the difference between the number of active members at the end of a particular period and the number of active members at the end of the previous period, divided by the average of active members at the end of a particular period and active numbers at the end of the previous period (the midpoint formula) [46]. The average annual continuous growth rate for the 8-year period was also calculated by dividing the number of active members at the end of 2017 by the number of active members at the end of 2010 to get the overall growth factor. We took a natural log of the growth factor to obtain the overall growth rate. This result was then divided by 8 to get the average annual growth rate for the study period [46]. Point and interval estimates (95% confidence intervals) for new enrolment and renewal of membership were also estimated by geographical region and member group to test for statistically significant differences [47]. All the analyses were performed using Microsoft excel 2013 version.

## Results

### Characteristics of the membership data

Table 1 summarizes percentage distribution of members of the scheme by category over the 8-year period, 2010–2017. It shows that persons below the age of 18 years were the most registered in each year of the study period, 48% in 2010 to 45.4% in 2017; followed by workers in the informal sector of the economy, about 32% to 30% over the same period. The proportion of indigent (extreme poor) short up remarkably from 1.4% to 14.2% between 2010 and 2014. The least group represented in the scheme was the security personnel. The NHIS started registering the security personnel in 2013; thus, there was no enrolment data for them in the first three years of the study period.

**Table 1:** Percentage distribution of members by category, 2010–2017

Membership category	Percentage of members							
	2010	2011	2012	2013	2014	2015	2016	2017
All categories	100.0	100.0	100.0	100.0	100.0	100.0	100	100
Informal sector workers	31.8	36.4	32.7	31.2	30.7	29.1	28.2	29.8
SSNIT contributors	4.7	4.5	3.8	3.3	3.5	3.9	4.7	5.4
SSNIT Pensioners	0.4	0.3	0.3	0.2	0.2	0.2	0.2	0.2
Persons below 18 years	47.7	49.7	47.2	43.2	44.9	44.1	41.4	45.4
Aged (≥70 years)	5.4	4.9	4.1	3.5	3.6	3.9	4.4	4.7
Indigent	1.4	4.2	4.1	11.3	14.2	13.1	13.9	6.5
Pregnant women	8.6	NA	7.8	7.1	2.7	5.7	7.1	7.9
Security personnel	NA	NA	NA	0.2	0.1	0.1	0.2	0.1
Total members	8,163,714	8,170,888	8,885,757	10,144,527	10,545,428	11,341,021	11,029,339	10,273,020

*SSNIT* Social Security and National Insurance Trust, *NA* not available

### Population coverage

**Fig. 2:**
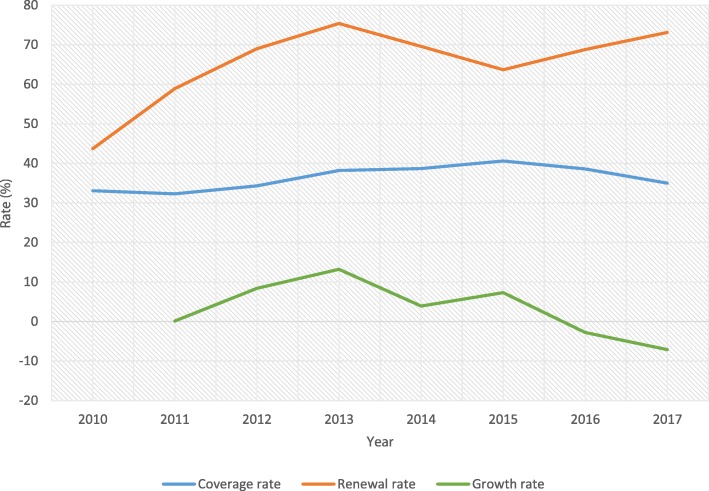
Trends in population coverage, renewal and growth rates

The population that enrolled in the scheme over the 8-year period increased from 33% (8.2 million) to about 41% (11.3 million) between 2010 and 2015 and declined to 35% (10.3 million) in 2017 (Fig. 2). Similarly, the members that renewed their memberships increased from 44 to 75.4% between 2010 and 2013 and then decreased to 64% in 2015 before rising to 73% in 2017. Over the study period, an average annual growth rate of about 3% was recorded. The highest growth of membership (13.2%) occurred in 2013 and declined thereafter to negative 7.1% in 2017.

### Enrolment by geographical region

**Fig. 3:**
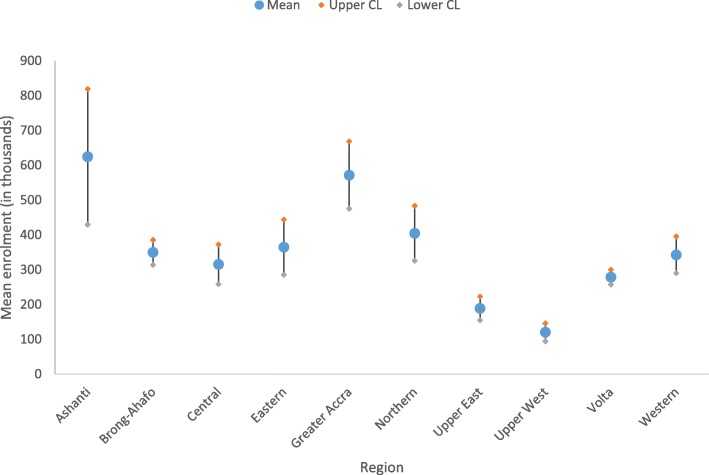
Point and interval estimates (95% CI) for new enrolment by region, 2010–2016

The average number of individuals who enrolled in the scheme as new members in the Ashanti region (M = 624,425; 95% CI: 429,714-819,136) over the 2010–2016 period was significantly higher than those in the other regions except Eastern, Greater Accra, and Northern (Fig. 3). Also, the average number of individuals in the Greater Accra region (M = 571,562; 95% CI: 474,912-668,211) who enrolled in the scheme was significantly higher than those in the other regions except Ashanti and Northern. Average number of members who enrolled in the scheme in Upper West was the lowest (M = 120,197; 95% CI: 94,088-146,307) among the 10 regions.

**Fig. 4:**
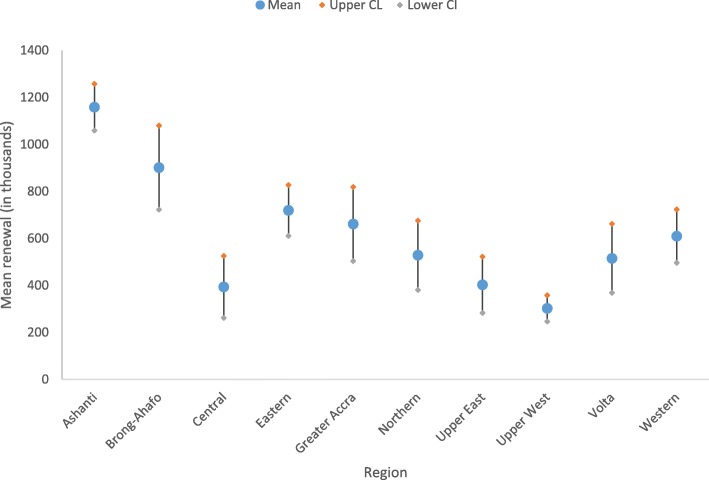
Point and interval estimates (95% CI) for membership renewal by region, 2010–2016. SSNIT: social security and national insurance trust; CL: confidence limits

Likewise, the average number of individuals who retained membership in the scheme was highest among those in the Ashanti region (M = 1,158,369, 95% CI: 1058644–1,258,095) and significantly higher than those in the other regions except Brong-Ahafo (Fig. 4). Again, the Upper West region recorded the lowest average number of members who renewed their memberships in the scheme (302,500; 95% CI: 246,600-358,400) but was significantly not different from those who renewed their memberships in Upper East (M = 402,659; 95% CI: 282,803-522,516) and Central (M = 393,818; 95% CI: 262,191-525,445) regions.

### Enrolment by member group

**Fig. 5:**
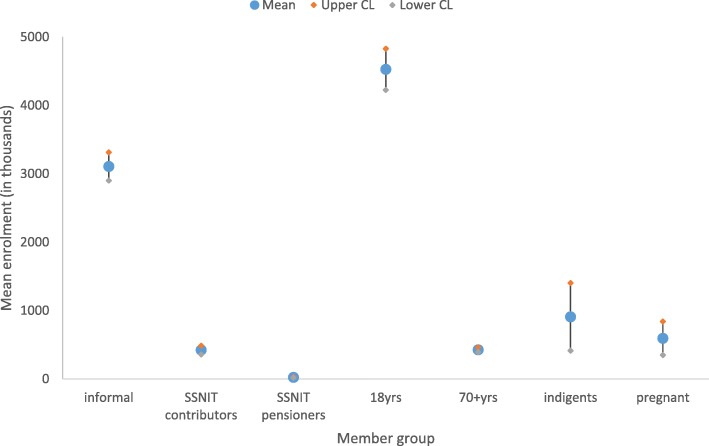
Point and interval estimates (95% CI) for enrolment by member category, 2010–2017 SSNIT: social security and national insurance trust; CL: confidence limits

The various member groups had no disaggregated data for new and renewed members. However, Fig. 5 shows that the average number of persons below the age of 18 years who enrolled in the scheme from 2010 to 2017 were significantly higher (M = 4,527,055; 95% CI: 4,224,883-4,829,227) than any other member group, followed by the informal sector workers (M = 3,106,788; 95% CI: 2,900,785-3,312,792). The SSNIT pensioners recorded the lowest average number of enrolment (M = 23,368; 95% CI: 19,072-27,664) over the study period.

## Discussion

Examination of the trends and characteristics of enrolment in the NHIS over the last eight years shows a decreasing trend of enrolment with significant variations among geographical regions and member groups. In the first four years of the study period, 2010–2013, there was a general increase in the number of individuals enrolling in the scheme and those renewing their memberships. These resulted in a steady growth in membership over the same period. Beyond 2013, the proportion of the population enrolling in the scheme showed an upward trend up to the year 2015 and declined consistently thereafter. However, the number of members renewing their memberships assumed a downward trend after the first four years of the study period and growth in membership declined remarkably over the same period.

The findings may be attributed to the voluntary participation in the scheme as explained by the conceptual framework for this study [33] In such SHI programmes or schemes, individuals are more willing to join at the initial stages, hoping to receive the benefits promised [33]. Therefore, apathy sets in if the benefits promised are below expectation of the members. Aside this natural tendency, other probable reasons for the decreasing trend of enrolment and renewal of membership are lack of knowledge of the scheme resulting from ineffective education and sensitization programmes, religious and cultural norms and poverty [30, 33]. Other plausible reasons are systemic factors including lengthy waiting times at registration centres, occasional shortage of registration materials, lengthy payment of provider claims and perceived poor quality of healthcare provider services. Many studies have found these factors to be associated with low enrolment and renewals in the scheme [29, 30, 34, 37, 48]. It is also possible that members of the scheme have a better alternative risk protection against their healthcare cost, for example, they may prefer other health protection schemes or out-of-pocket payment due to perceived poor quality of care for NHIS card-holders.

Although the NHIS has a one month waiting period for first time enrolees, the downward trend in enrolment may indicate adverse selection, a situation where individuals only enrolled in the scheme when they need healthcare services and refuse to renew their membership after receiving care. Evidence shows that insurance programmes with low participation and high turnover are more likely to suffer from adverse selection, leading to high claims payment, increased administrative expenses and reduced revenues [27, 33]. The pregnant women are more likely to indulge in this unhealthy practice because they are exempted from paying contribution to the scheme. Thus, there is an increased incentive for them to enrol when they get pregnant and refuse to renew their membership after delivery. This situation is a challenge in most SHIs and could threaten sustainability when left unaddressed because it reduces revenue and risk pooling. Our findings are consistent with a number of studies on the NHIS [24, 31, 37].

Findings of the study also show significant differences in enrolment and renewal among the ten administrative regions of the country. Individuals in the predominantly urban regions such as Ashanti and Greater Accra, are significantly enrolling in the scheme more than any other region. This trend of enrolment may be attributed to a number of factors including population density and availability of healthcare facilities and health workforce. The more urbanised regions have higher population; thus, may have higher number of individuals who were not previously enrolled in the scheme compared to the less densely populated regions such as Upper East and Upper West. In addition, there are more healthcare facilities and professionals, both in the public and private sectors in the two urbanised regions than any other region. Evidence shows that availability of these resources improves enrolment by providing geographical access to healthcare services [24, 30, 34]. Besides, individuals living in these two largely urbanised regions could afford to pay the NHIS premium due to improved economic opportunities and relatively low incidence of poverty. The findings support studies by Van der Wielen [49] and Dake [50] which show higher NHIS coverage of individuals living in urban areas than those in the rural areas.

The two regions, Upper East and Upper West which recorded significantly lower enrolment of new members over the study period are predominantly more rural; thus, have less healthcare infrastructure and health professionals, high incidence of poverty and low level of education. These factors serve as barriers to enrolment [18, 24, 34, 35, 51]. There is also evidence of strong religious and cultural norms in these regions that mitigate against enrolment in the NHIS, for example, some of the women may have to seek consent from their spouses before they can take certain personal decisions such as visiting healthcare facilities for treatment [30, 51, 52]. Our findings contrast that of past trend analyses, where individuals in the largely rural regions such as Upper East, Upper West, Western, Central and Volta enrolled in the scheme more than those in the urbanised regions, Ashanti and Greater Accra [17, 28]. Probably, this is due to dissatisfaction of services with the scheme and healthcare providers as shown in other studies [24, 34–36]. However, the significant differences in enrolment among the geographical regions are consistent with similar studies [17, 28].

Our study also shows significant variations in enrolment among the defined member groups of the NHIS. Persons below the age of 18 years and workers in the informal sector of the economy are significantly enrolling in the scheme more than any other member group. The higher number of persons below the age of 18 years in the scheme is understandable; they are more susceptible to diseases and therefore more likely to enrol for protection against their healthcare cost. Also, persons below the age of 18 years are exempted from paying premium and this serves as an incentive for their parents to enrol them in the scheme. Although this trend is good for achieving UHC [53], there would be a need for adequate subsidies to cater for the expected increase in provider claims cost to the scheme and prevent sustainability threat because persons below the age of 18 years are more likely to utilize healthcare services and incur higher cost than the other member groups.

The significantly high enrolment of the informal sector group may be due to their higher proportion in the employment sector and lack of social security for them, especially social health protection. This group constitutes about 80% of the workforce in the country and face a number of risks including lack of unemployment insurance, employment-based health insurance and statutory pensions. Thus, weighing the cost-benefit combination of enrolling in the NHIS, majority of them would make a rational decision to join the scheme. The high enrolment of the informal sector group is also encouraging because it would boost revenue and ensure maximum risk pooling for long term viability of the scheme. The findings also show that the poor and vulnerable groups such as indigents, the aged, SSNIT pensioners and the pregnant women are less enrolled, consistent with a number of studies [18, 25, 31, 52]. The significant differences in enrolment among the member groups also corroborate other studies [28, 31].

Our findings suggest the need for enforcement of the mandatory enrolment stipulated in the National Health Insurance Act (ACT 852 of 2012) to increase enrolment and enlarge the risk pool. This can be done by making the NHIS card a pre-requisite for obtaining or acquiring certain services, for example, driver license, employment in public and private sectors, etc. as is being done with enrolment in second cycle and tertiary educational institutions in the country. To address the anticipated claims cost from increased enrolment, the regulator (the NHIA) could rally support from civil society organisations or groups for the proposed increment in the health insurance levy and SSNIT contribution deductions and levy on tobacco and sugar sweetened drinks. Alternatively, the NHIA could propose to government to decouple the health insurance levy from the VAT and lodge the money directly into the National Health Insurance Fund (NHIF). This would ensure readily available funds for payment of healthcare provider claims and continuous provision of services to the NHIS members.

### Limitations

This study has a number of data limitations. First, disaggregated data by geographical region for the year 2017 were not available at the time of the study. Secondly, there were no disaggregated membership data on sex; thus, the study could not examine trends and characteristics of enrolment for male and females. Likewise, there were no disaggregated data on renewals for member groups, making it difficult to show which groups of the members are mostly renewing their membership. Nonetheless, the analysis of active membership by member groups provides overview of groups that are mostly enrolling in the scheme. Moreover, there were no data on members who have passed on during the years under study and have become ineligible to renew their membership. This situation is likely to distort the true picture of the number of active members renewing their membership because the number of potential renewals, which is the denominator in the renewal ratio formula would increase whilst the number of renewals (numerator) remains constant, thereby resulting in a lower renewal rate.

## Conclusions

The study reveals that enrolment in the NHIS is  declining and there are significant differences among the geographical regions of the country and member groups. Individuals in the urbanised regions are enrolling in the scheme more than those in the rural regions and persons below the age of 18 years and the informal sector workers are enrolling more than the other member groups. The decreasing trend in enrolment and significant disparities observed could reduce revenue and risk pooling and eventually compound the sustainability challenges facing the scheme. Innovative strategies such as the use of mobile phone application for registration and renewal would be useful for improving enrolment towards achieving UHC. Addressing supply-side challenges such as delays in payment of healthcare provider claims and unauthorized co-payments as found in several studies would also be necessary to improve satisfaction of the members and ensure continuous enrolment.

## Additional file

Additional file 1: Distribution of population by region, sex, and locality of enumeration, 2010. Source: Ghana Statistical Service [39]. (DOCX 62 kb)

## Abbreviations

CHPS: Community-based health planning and services
ENT: Ear, nose and throat
GHS: Ghana cedi
LEAP: Livelihood empowerment against poverty
LMIC: Low and middle-income country
NHIA: National health insurance authority
NHIF: National health insurance fund
NHIS: National health insurance scheme
PHC: Population and housing census
SSA: Sub-saharan africa
SSNIT: Social security and national insurance trust
UHC: Universal health coverage
VAT: Value-added tax
