# Supplementary material for: Trends and characteristics of enrolment in the National Health Insurance Scheme in Ghana: a quantitative analysis of longitudinal data
Source: Glob Health Res Policy. 2018 Nov 13;3:32. doi: 10.1186/s41256-018-0087-6 (PMC6233555; doi:10.1186/s41256-018-0087-6)
Supplement: Supplementary file 1 — Distribution of population by region, sex, and locality of enumeration, 2010. Source: Ghana Statistical Service [39]. (DOCX 62 kb) [file 41256_2018_87_MOESM1_ESM.docx]

**Additional file 1**: Distribution of population by region, sex, and locality of enumeration, 2010

**Source**: Ghana Statistical Service [36]
